# Proteomics investigation identifies prominent changes in synapse-related proteins in a fragile X mouse model

**DOI:** 10.1186/1471-2202-16-S1-P21

**Published:** 2015-12-18

**Authors:** Jantine AC Broek, Z Lin, H Van't Spijker, S Ozcan, HM De Gruiter, ED Haasdijk, R Willemsen, CI De Zeeuw, S Bahn

**Affiliations:** 1 Dept. of Chemical Engineering and Biotechnology, University of Cambridge, Cambridge, UK; 2 Erasmus Optical Imaging Center, Erasmus MC, Rotterdam, the Netherlands; 3 Dept. of Neurosciences, Erasmus MC, Rotterdam, the Netherlands; 4 Dept. of Clinical Genetics, Erasmus MC, Rotterdam, the Netherlands; 5 Netherlands Institute for Neurosciences, Royal Academy for Arts and Sciences, Amsterdam, the Netherlands

## 

Fragile X syndrome (FXS) is a single gene disorder that is the most common heritable cause of intellectual disability and the most frequent monogenic cause of autism spectrum disorders (ASD). FXS is caused by trinucleotide repeats in the promoter region of the fragile X mental retardation gene (*fmr1*). This leads to the downregulation of the fragile X mental retardation protein (FMRP), which regulates translation of a wide range of proteins. The extent of expression level alterations of synaptic proteins affected by FMRP loss and their consequences on synaptic dynamics in FXS has not been fully investigated. Here, we have investigated the molecular mechanisms underlying FXS by identifying the molecular signature and elucidating the function of FXS-associated proteins. The first part of the study consists of an exploratory molecular profiling study on brain tissue samples of a *fmr1 *KO mouse model using shotgun label-free liquid chromatography mass spectrometry (LC-MS^E^). These studies indicate that most of the changed proteins are involved in synaptic functions. Thus, as a next step we investigated synaptosomes from the cerebellum and hippocampus using LC-MS^E ^and label-based selected reaction monitoring (SRM). Key findings relate to altered levels of proteins involved in GABA-signalling in the cerebellum. To explore these findings further, FM1-43 dye was used in cultured hippocampal neurons and cerebellar Purkinje cells to track vesicle recycling and unloading profiles. Furthermore, ultrastructural analysis of synaptic vesicles of the GABA-ergic Purkinje cells in the cerebellum was performed to investigate potential pre-synaptic effects. Taken together, these studies provide novel insights into the molecular changes associated with FXS.

